# Diffuse, IDH-wildtype gliomas in adults with minimal histological change and isolated *TERT* promoter mutation: not simply CNS WHO grade 4

**DOI:** 10.1007/s00401-024-02773-3

**Published:** 2024-07-29

**Authors:** L. P. Priesterbach-Ackley, F. Cordier, P. de Witt Hamer, T. J. Snijders, P. A. Robe, B. Küsters, W. W. J. de Leng, W. F. A. den Dunnen, D. Brandsma, C. Jansen, P. Wesseling, A. Muhlebner

**Affiliations:** 1 Department of Pathology, University Medical Center Utrecht, Internal Mail No. H04.312, P.O. Box 85500, 3508 GA Utrecht, The Netherlands; 2 Department of Pathology, Ghent University Hospital, Ghent, Belgium; 3 Department of Neurosurgery, Amsterdam University Medical Centers/VUmc and Brain Tumor Center, Amsterdam, The Netherlands; 4 Department of Neurology and Neurosurgery, Brain Center Rudolf Magnus, University Medical Center Utrecht, Utrecht University, Utrecht, Utrecht, The Netherlands; 5 Department of Pathology, Radboudumc, Nijmegen, The Netherlands; 6 Department of Pathology, University Medical Center Groningen, Groningen, The Netherlands; 7 Department of Neuro-Oncology, Netherlands Cancer Institute, Amsterdam, The Netherlands; 8 Laboratory for Pathology Eastern Netherlands, LABPON, Hengelo, The Netherlands; 9 Department of Pathology, Amsterdam University Medical Centers/VUmc and Brain Tumor Center Amsterdam, and Princess Máxima Center for Pediatric Oncology, Utrecht, The Netherlands

The 5th edition of the WHO Classification of CNS Tumors requires molecular testing for certain entities to achieve a precise pathologic diagnosis [5]. In this case series, we aimed to investigate the extent to which the molecular features consistent with the diagnosis of ‘molecular glioblastoma’ (m-GBM) [5] correlate with overall survival (OS) in cases suspected of a diffuse low-grade glioma based on radiologic and histologic examination. In the presented cases, a definite diagnosis of m-GBM was reached based on the current WHO 2021 criteria, but caution is warranted when molecular aberrations are limited.

We compiled a cohort of individual, non-consecutive clinical cases, between 2017 and the end of July 2023. These cases were collected from four different centers in the Netherlands. The inclusion criteria required cases to have radiologic features suggestive of a low-grade diffuse glioma on MRI, and Hematoxylin and Eosin (H&E) stained tissue slides from biopsy or resection brain samples showing subtle features consistent with a low-grade diffuse glioma. Children and cases showing high-grade histologic features (e.g., brisk mitotic activity, florid microvascular proliferation and/or necrosis (according to WHO guidelines)) or high-grade radiologic features (e.g., ring-like contrast enhancement) were excluded. Molecular analysis of these samples was performed using next-generation sequencing (NGS) [4] (*n* = 56) and methylation profiling (MP) with the Brain Tumor Classifier (v12.5 or v12.8 [2]) in a subset of these cases (*n* = 23). Cases with H3 p.K28 (p.K27) or IDH mutations were not included in the analysis.

OS was determined as the interval from initial radiologic diagnosis to last contact or death. Kaplan–Meier estimates, Log-Rank tests and Hazard Ratio (HR) of the OS were calculated using SPSS v.29 (IBM). The patients presented in this case series had a median age of 62 years with a range from 25 to 80 years. Thirty-five were male and 21 were female. Follow-up of the patients varied from 1 month (deceased after 1 month) to 6 years (median follow up 15 months). At the end of follow-up (July 2024), 20 patients were still alive.

**Fig. 1:**
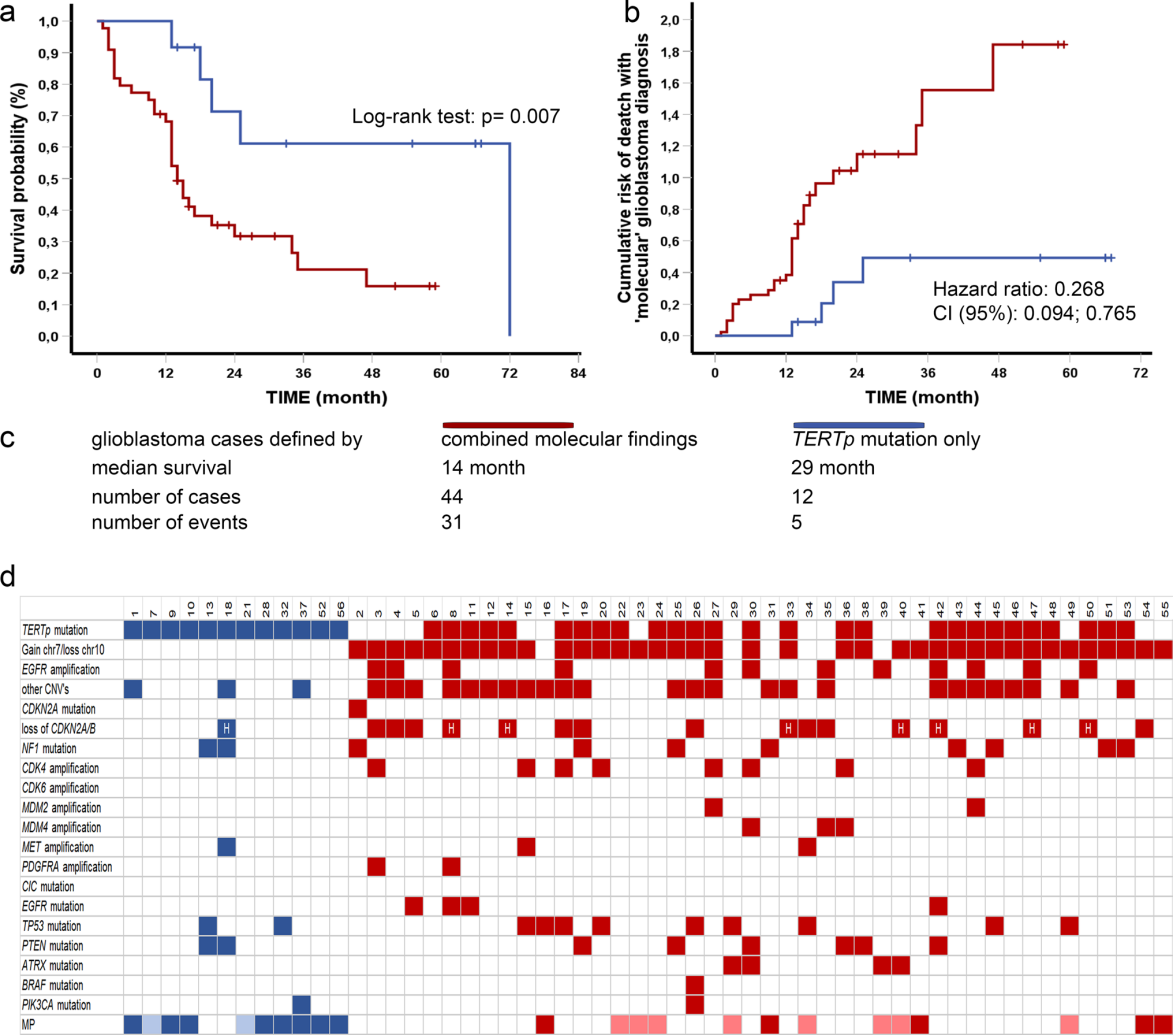
Overall survival (OS) and cumulative hazard curves of patients within the m-GBM group; **a** OS was significantly better in patients with an isolated *TERT*p mutation (represented in blue; defined as absence of *EGFR* amplification and/or +7/−10; *TERT*p mutation only) than in patients with a *TERT*p mutation with an *EGFR* amplification and/or +7/−10 (represented in red; combined molecular findings). **b** Patients with a *TERT*p mutation only show a lower HR compared to patients with a *TERT*p mutation and an *EGFR* mutation and/or +7/−10. **c** Legend and descriptive data of the cohort. **d** Oncoplot showing all molecular aberrations detected using next generation sequencing and the results of methylation profiling (MP). Cases included in the ‘*TERT*p-only’ group are clustered and designated in *blue*, the other cases are labeled in *red*. H indicates homozygous loss; transparency indicates score under threshold of 0.9

Details on the molecular aberrations that were found are listed in Fig. 1c. In line with the WHO 2021 CNS tumor classification, presence of a *TERT* promoter (*TERT*p) mutation, *EGFR* amplification and/or combination of gain of complete chromosome 7 (+7) and loss of complete chromosome 10 (−10) were considered molecular high-grade features. A *TERT*p mutation was identified in 39 cases, and in 12 of those it was not accompanied by *EGFR* amplification and/or the combination of +7/−10 chromosomal aberrations. Clinical information of these 12 ‘*TERT*p-only’ cases are provided in Supplementary Table 1. Other molecular aberrations that were found but that are not used as criteria for m-GBM were not taken into account in the further analysis.

Copy number variations compatible with m-GBM were found in 40 cases. Of these, 38 cases showed +7/−10 and 12 cases showed an *EGFR* amplification. Using MP, a calibrated score of >0.9 was obtained in 13 of the 23 cases, with rare methylation groups of high-grade diffuse glioma in 7 cases (Supplementary Table 1). These latter groups consisted of methylation classes adult-type diffuse high-grade glioma, IDH-wildtype, subtypes B, E and F [3]. Of the 12 ‘*TERTp*-only’ cases, 10 cases were submitted for methylation profiling. Of these, two cases had a calibrated score below the threshold of 0.9 and two cases were classified as ‘control tissue’. Considering the 6 cases with a score of 0.9 or higher, two were classified as ‘Adult-type diffuse glioma’, and within that group ‘Adult-type diffuse high-grade glioma, IDH-wildtype subtype F’, two were classified as ‘Glioblastoma, pediatric type’, another one matched with ‘Adult-type diffuse high-grade glioma, IDH-wildtype, subtype B’ and only one case was classified as ‘Glioblastoma, IDH-wildtype’.

Clinical follow-up data were available for all 56 patients. We compared the OS in patients with m-GBM and an isolated *TERT*p mutation (disregarding any additional molecular aberrations that do not contribute to the diagnosis of m-GBM) with those with a *TERT*p mutation and additional amplification of *EGFR* and/or +7/−10. Cases with m-GBM based on *TERT*p mutation only demonstrated a significantly longer OS (Log-Rank test *p* = 0.007, see Fig. 1a). Additional Cox regression analysis revealed that the cumulative risk of death is lower in the m-GBM group with isolated *TERT*p mutation as compared to the m-GBM group with both a *TERT*p mutation and/or *EGFR* amplification and/or +7/−10 alterations (HR = 0.27; CI 95%: 0.09–0.77; see Fig. 1b). Moreover, the presence of a *TERT*p mutation along with +7 (but without −10 or *EGFR* amplification) was associated with a worse prognosis (HR = 2.172; CI 95%: 1.03–4.70). Adjustment for age did not influence the results.

According to the WHO 2021 classification, the presence of a *TERT*p mutation in the context of an IDH-wildtype diffuse astrocytoma suggests clinical behavior resembling glioblastoma. These *TERT*p mutations often occur together with +7/−10, as was true for 27 cases in this series. However, even without these additional molecular features, the presence of a *TERT*p mutation indicates unfavorable clinical behavior. In contrast to recent studies [6, 7], in our series we observed better OS in cases with only *TERT*p mutations compared to those with *TERT*p mutations accompanied by *EGFR* and/or +7/−10 alterations. On average, the cases in the ‘*TERT*p-only’ group showed less concomitant genetic alterations compared to the other m-GBM cases.

For the cases in this series, an integrated final diagnosis was established following multidisciplinary discussions, to guide treatment- and follow-up decisions. Careful clinico-pathologic correlation is crucial to assess the actual clinical behavior of each tumor, considering the possibility of lead time bias due to early detection or undersampling [8]. Although, previous studies suggested that a *TERT*p mutation in a sample with radiologic and histologic features of a diffuse glioma may be sufficient to diagnose m-GBM [6, 7], our findings corroborate the results of Berzero et al. and underscore the need for caution in cases that have low-grade histology and isolated *TERT*p only [1]. The prognosis of these cases differs from those with additional genetic aberrations commonly associated with high-grade behavior. Thus, these tumors seem to be different from m-GBM cases with +7/−10 and/or *EGFR* amplification, but may or may not constitute a separate group of their own. Future research should elucidate the clinical parameters of gliomas characterized by *TERT*p only, and whether the prognosis is more similar to WHO grade 3 clinical behavior. Samples with only *TERT*p mutations might require additional MP to identify ‘rare subtypes’ of adult-type diffuse high-grade glioma, IDH-wildtype, as defined by the Heidelberg Brain Tumor classifier, which are linked to a more favorable prognosis [3].

### Supplementary Information

Below is the link to the electronic supplementary material.

Supplementary file1 (DOCX 16 KB)
