# Supplementary material for: Diffuse, IDH-wildtype gliomas in adults with minimal histological change and isolated TERT promoter mutation: not simply CNS WHO grade 4
Source: Acta Neuropathol. 2024 Jul 29;148(1):12. doi: 10.1007/s00401-024-02773-3 (PMC11286727; doi:10.1007/s00401-024-02773-3)
Supplement: Supplementary file 1 — Supplementary file1 (DOCX 16 KB) [file 401_2024_2773_MOESM1_ESM.docx]

**Supplementary Table 1. Clinical information and methylation profiling results of all ‘*TERT*p-only’ cases (blue) together with mGBM-cases with a calibrated score above >0.9 for methylation profiling (red).**

| **Case number** | **Age/sex** | **Location** | **Match** | **Calibrated score** | **Classifier version** |
| --- | --- | --- | --- | --- | --- |
| 1 | 26/M | Frontal left | Glioblastoma, pediatric type, RTK1, subclass A | 0.99 | V12.8 |
| 7 | 53/M | Parietal right | Ependymal tumors | 0.3 | V12.8 |
| 9 | 68/M | Temperoinsular left | Adult type diffuse glioma, subtype: diffuse high-grade neuroepithelial tumour (adult type, non-defined type D), subtype: adult type diffuse high grade glioma, IDH-wildtype, subtype F | 0.99 | V12.8 |
| 10 | 73/F | Left hemisphere, multifocal | Control tissue corpus callosum | 0.99 | V12.8 |
| 13 | 29/F | Temporal left | Not executed |  |  |
| 16 | 73/M | Pons | Glioblastoma, IDH-wildtype, RTK1 | 0.99 | V12.8 |
| 18 | 67/M | Periventricular and temporal left | Not executed |  |  |
| 21 | 62/M | Brainstem and cerebellum | Control tissue of the hemispheric cortex | 0.64 | V12.8 |
| 28 | 66/M | Frontal left | Control tissue corpus callosum | 0.99 | V12.8 |
| 31 | 29/F | Frontal right | Adult-type diffuse high grade glioma, IDH-wildtype, subtype B | 0.99 | V12.5 |
| 32 | 27/M | Frontal right | Adult-type diffuse high grade glioma, IDH-wildtype, subtype B | 0.99 | V12.5 |
| 37 | 25/F | Pons, cerebellar peduncle | Glioblastoma, pediatric type, not otherwise specified, subtype B | 0.99 | V12.5 |
| 41 | 58/F | Frontal left | High grade neuroepithelial tumor, NOS, subtype E | 0.94 | V12.5 |
| 52 | 77/M | Temporal right | Adult type diffuse glioma, subtype: diffuse high-grade neuroepithelial tumour (adult type, non-defined type D), subtype: adult type diffuse high grade glioma, IDH-wildtype, subtype F | 0.99 | V12.5 |
| 54 | 64/M | Parietal left | Adult type diffuse glioma, subtype: diffuse high-grade neuroepithelial tumour (adult type, non-defined type D) (0,98), subtype: adult type diffuse high grade glioma, IDH-wildtype, subtype F (0,98) | 0.99 | V12.5 |
| 55 | 79/F | Temporal left | Adult type diffuse glioma, subtype: diffuse high-grade neuroepithelial tumour (adult type, non-defined type D) (0,98), subtype: adult type diffuse high grade glioma, IDH-wildtype, subtype F (0,98) | 0.99 | V12.5 |
| 56 | 78/M | Parietal left | Glioblastoma, IDH-wildtype | 0.92 | V12.5 |
